# A survey of facilitators and barriers to recruitment to the MAGNETIC trial

**DOI:** 10.1186/s13063-016-1724-3

**Published:** 2016-12-23

**Authors:** Geetinder Kaur, Rosalind L. Smyth, Colin V. E. Powell, Paula Williamson

**Affiliations:** 1 School of Health and Population Sciences, University of Birmingham, Edgbaston, Birmingham, B15 2TT UK; 2 University College London, 30 Guilford Street, London, WC1N 1EH UK; 3 Division of Population Medicine, Cardiff University School of Medicine, UHW Main Building, Heath Park, Cardiff, CF144XN UK; 4 MRC North West Hub for Trials Methodology Research, Institute of Translational Medicine, University of Liverpool, Crown Street, Liverpool, L69 3BX UK

**Keywords:** Recruitment, Children, Clinical trials, RCT (randomised controlled trial), Survey

## Abstract

**Background:**

Recruitment to randomised controlled trials with children is challenging. It is imperative to understand the factors that boost or hinder recruitment of children to clinical trials. We conducted a survey of facilitators and barriers to recruitment to the MAGNETIC trial, using a previously developed web-based tool.

**Methods:**

MAGNETIC is a multicentre randomised trial of nebulised magnesium in acute severe asthma, recruiting 508 children from 30 UK sites. Recruiters were asked to grade a list of factors from –3 to +3 depending on whether the factor was perceived as a strong, intermediate or weak barrier (–3 to –1) or facilitator (+1 to + 3), and using (0) if it was thought to be not applicable. Free text responses were invited on strategies applied to counter the identified barriers.

**Results:**

The commonly identified facilitators were motivation and experience of study teams, effective communication and coordination between teams at site and between sites and the Clinical Trials Unit, the presence of designated research nurses, good trial management, clinical trial publicity, simple inclusion criteria, effective communication with parents and presentation of trial information in a simple and clear manner. The commonly identified barriers were heavy clinical workload, shift patterns of work, Good Clinical Practice (GCP) training, inadequate number of trained staff, time and setting of consent seeking, non-availability of research staff out of hours and parents' concerns about their child taking an experimental medicine. Having a designated research nurse, arranging GCP training and trial-related training sessions for staff were the most commonly reported interventions.

**Conclusions:**

This study highlights important generic and trial-specific facilitators and barriers to recruitment to a paediatric trial in the acute setting and provides information on the recruitment strategies or interventions that were applied to overcome these barriers. This information can be very useful in informing the design and conduct of future clinical trials with children, particularly in the acute or emergency setting.

**Trial registration:**

ISRCTN, ISRCTN81456894. Registered on 15 November 2007.

**Electronic supplementary material:**

The online version of this article (doi:10.1186/s13063-016-1724-3) contains supplementary material, which is available to authorized users.

## Background

Conducting clinical trials with children is challenging. Protection from harm due to an intervention and obtaining consent or assent are particular challenges in paediatric studies [1, 2]. Recruiting adequate numbers of eligible participants is crucial for successful completion of a trial. Poor recruitment impacts the validity of study findings, is a common cause of requests for trial extensions and may result in premature termination of trials [3–5]. Recruitment to a randomised controlled trial can be affected by many internal and external factors. Understanding how these factors influence the success or failure of large multicentre studies and how clinical teams overcome them will improve the planning, design and conduct of future clinical trials [4]. We conducted a survey from all those sites that contributed to recruitment to a large multicentre study examining nebulised magnesium sulphate in acute asthma in children (the MAGNETIC study) [6]. The aim was to explore the facilitators and barriers to recruitment during that study.

## Methods

### Setting

The MAGNETIC trial was a randomised, multicentre, double-blind, placebo-controlled trial evaluating the role of nebulised magnesium in severe acute asthma in children unresponsive to standard inhaled treatment. Children aged from 2 to 16 years old, presenting to an emergency department or children’s assessment unit with acute severe asthma were given conventional treatment on presentation and reassessed after 20 minutes; those fulfilling the eligibility criteria were enrolled into the study. Written informed consent was obtained from parents or guardians in the 20-minute period when the child was receiving initial treatment [6].

The trial recruited successfully to target, enrolling 508 children from 30 sites across the UK, needing a short extension of 3 months beyond the planned recruitment period of 24 months. Despite the overall success of the trial, the recruitment experience at sites was variable. The sites were a mix of tertiary children’s hospitals with paediatric intensive care unit (PICU ) with extensive paediatric research experience (40%); medium (34%) and large (23%) district general hospitals with no PICU and minimal research experience; and large general hospitals with extensive research experience but less paediatric research experience (3%). There were three additional sites which had opened but could not recruit any patients and a further four where efforts were made to set up the trial but they did not open to recruitment. We conducted a survey of recruitment experience of clinical teams at all sites to explore their views on facilitators and barriers to recruitment and gather information on various interventions that were applied to overcome the hurdles that were identified at each site.

### Tool used

The survey was conducted using a previously developed, web-based recruitment survey tool which provides an evidence-based, comprehensive list of potential facilitators and barriers [7], using online survey software (www.surveygizmo.com). The survey questionnaire was divided into three sections to collect information on:

1. Responder characteristics such as name of site, study role and duration and period of involvement in the trial
2. Perception of clinical teams with regard to facilitators and barriers to recruitment
3. Recruitment strategies or interventions that were applied at various sites to improve recruitment.

The perception of clinical teams with regard to facilitators and barriers to recruitment was assessed by asking the responders to score a preformed, evidence-based list of potential factors that affected recruitment to clinical trials [7]. These factors were categorised in terms of operating at the level of trial, site, patient, clinical team, information and consent process and central study team.

The responders were asked to grade each factor from –3 to +3 depending on whether the factor was perceived as a strong (–3), intermediate (–2) or weak (–1) barrier, or a weak (+1), intermediate (+2) or strong (+3) facilitator or (0) if the factor was thought to be not applicable. Open questions were asked to gather information on interventions and strategies applied by clinical teams to counter the problems that were identified at the sites (see the Appendix, Table 5).

### Who was asked?

The survey was conducted from August 2011 to February 2012, following completion of trial recruitment at all sites. Permission to contact the clinical staff was obtained from the Principal Investigator (PI) at each site, following which their contact details were requested from the National Institute of Health Research (NIHR) Medicines for Children Research Network (MCRN) Clinical Trials Unit, which was responsible for coordinating the trial. The link to the online survey was emailed to clinical teams involved with recruitment to the trial. The list of available contacts consisted of medical and nursing staff delegated to recruit to the trial. This included the PIs, research nurses, medical practitioners, nurse practitioners and nursing staff. No formal sample size calculation was done. The survey questionnaire was emailed to all available contacts, but extra efforts were made to obtain responses from PIs and at least one research nurse at each site. This was done to gather the views of a stable research team engaged with recruitment at each site, as trainee doctors and nurses were expected to change over and move between hospitals over the 2-year study recruitment period.

An initial invitation describing the aims of the survey provided the link for completion. Voluntary participation was requested, and potential responders were reassured that no personal information would be collected, no sites would be identified in any publication and confidentiality of data would be maintained. The non-responders were sent two subsequent email reminders spaced 4 weeks apart. The PIs and research nurses were sent additional email reminders followed by a telephone reminder.

Commonly identified facilitators and barriers were defined a priori as those that were identified as a facilitator or barrier by 50% or more of the responders. Responders who scored a factor as –1, –2 or –3 were aggregated to give the total number of responders who identified the factor as a barrier. Similarly, responders who scored a factor as +1, +2 or +3 were added to give the total number of responders who identified the factor as a facilitator. In addition to the overall responses, the PI and research nurse responses were analysed separately, and the total number of responders who identified the factor as facilitator or barrier or not applicable were calculated in a similar manner. One PI and one research nurse response per site, chosen at random, were included in the analysis to ensure equal representation of sites. The free text responses on recruitment strategies were grouped into recurring themes using the NVivo 10 qualitative data package.

## Results

A list of 656 potential contacts was obtained from the study delegation log of which contact details could be obtained for 491. This included PIs and research nurses at all 37 sites and other clinical staff at 30 of the 33 open sites; permission to contact other staff could not be obtained from the PIs at the remaining 3 sites.

A total of 206 responses were received: 169 complete and 37 partial responses. Of the 37 partial responses, 14 were duplicate responses, no data were recorded in 20 and less than 25% of the questions were answered in 3. These were excluded from analysis. The number and percentage of overall responses by role, duration and period of involvement are shown in Table 1. The overall response rate to the survey was 39%; 192 of 491 responses were received after excluding the duplicate responses. The response rates for PIs and at least one research nurse per site are presented in Table 2. Each site was represented by the PI and research nurse. The commonly identified facilitators and barriers to recruitment identified in overall responses are ranked in order of frequency and presented in Table 3.

**Table 1:** Number (%) of responses by role, duration and period of involvement

	*n*	%
Role (*n* = 169)		
PIs	33	19.5
Medical practitioners	71	42
Research nurses	42	24.9
Others^a^	23	13.6
Duration of involvement (*n* = 169)		
Whole trial period	92	54.4
Part of trial period^b^	75	44.4
No response	2	1.2
^b^Period of involvement (*n* = 75)		
Set-up/early recruitment period	14	18.7
Once trial established at site	54	72
Both	3	4
No response	4	5.3

^a^Others included staff nurses on ward, day units and accident and emergency departments, paediatric nurse practioners and nurse manager

^b^Period of involvement for responders who were not involved for the whole trial period

**Table 2:** Response rates for PIs and at least one research nurse per site

Sites	Number of PIs	PI responses	Number of research nurses	RN responses (one per site)
Sites that recruited (*n* = 30)	29*	27 (93%)	28 ^α^	28 (100%)
Sites that opened but didn’t recruit (*n* = 3)	3	3 (100%)	1 ^α, β^	1 (100%)
Sites that never opened (*n* = 4)	4	2 (50%)	1 ^β^	1 (100%)

*PI at one site on maternity leave

*α* RN had left post before the survey was conducted

*β* No designated research nurse at site

**Table 3:** Facilitators and barriers to recruitment in order of frequency of responses

Facilitators	*n* (%)	Barriers	*n* (%)
Motivation of MAGNETIC study team at site	131 (78.9)	Clinical workload	146 (87.3)
Communication and coordination between study team members at site	123 (74.5)	Shift patterns of work	129 (77.7)
Communication skills of clinical team	116 (70.3)	Number of trained staff	130 (77.3)
Presence of designated research nurse/practitioner	113 (68.1)	Time and setting of consent seeking	127 (76)
Research experience of PI and study team members at site	105 (63.3)	GCP training	116 (69.6)
Publicity by the trial team	103 (62.9)	Time to complete administrative work related to the trial	112 (66.6)
Communication and coordination between study team at site and Clinical Trials Unit (CTU)	103 (62.1)	Parents’ concerns about side effects of new drug	109 (65.3)
Trial management	101 (62)	Parents’ attitude towards their child taking experimental medicine or placebo	95 (57.2)
Clinician attitude to involving patients in research	101 (60.9)	Availability of research staff out of hours	95 (57)
Perceived importance of the particular research question	99 (60.1)	Difficulty in approaching patients for consent	90 (53.9)
Availability of designated research team	97 (58.4)	Local clinical arrangements	86 (52.1)
Clarity in presentation of trial information	97 (58.4)	Seasonal variation	86 (51.8)
Patient inclusion criteria	96 (57.5)	Language or cultural barriers	84 (50.3)
Motivation of clinical team	89 (53.6)	Amount and complexity of trial information provided	83 (50)
Experience and training of clinical team seeking consent	84 (50.4)		
Communication between research team and parents	83 (50.1)		

### Facilitators of recruitment

Motivation and commitment of the study team was the most commonly identified facilitator to trial recruitment (78.9%). Effective communication and coordination between study team members at the site (74.5%) and between the site and the Clinical Trials Unit (CTU) were also recognised to be very important. The presence of a research nurse (68.1%) and having a designated research team (58.4%) were thought to be very helpful in assisting busy clinical teams with trial recruitment and data collection. An experienced Principal Investigator (PI) (63.3%) and an enthusiastic clinical team with good communication skills (70.3%) were thought to be instrumental in resolving local issues and ensuring successful trial recruitment at sites. The clinical team’s perception of the importance of the research question (60.1%) and a positive attitude to involving patients in research (60.9%) were felt to be very important. Encouragement and support provided by PIs, senior clinicians and research nurses was important to keep up the motivation levels of staff and develop a positive research culture. Trial management support (62%) and effective communication between the CTU and study teams at sites (62.1%) were recognised as facilitators. Internal trial publicity by the study teams (62.9%) helped to maintain the presence of the MAGNETIC trial among teams and increase parents’ and families’ awareness about the trial. Simple patient inclusion criteria (57.5%) and clear presentation of trial information (58.4%) boosted recruitment. Good communication between research teams and parents (50.1%) and consent seeking by experienced and trained clinicians (50.4%) were thought to be very helpful in overcoming barriers such as parental anxiety about the potential adverse effects of the trial drug and their child taking an experimental medication or placebo.

### Barriers to recruitment

Parents’ concerns about side effects (65.3%) and their attitude towards their child taking experimental medicine or placebo (57.2%) were recognised as important barriers. Clinical teams expressed discomfort in approaching patients to seek consent (53.9%). Excessive amounts and complexity of trial information and the time taken to complete trial-related administrative work were criticised (66.6%). Additionally, language and cultural barriers were thought to hinder recruitment (50.3%). Recruitment difficulties arising due to seasonal variation were also recognised (51.8%).

Heavy clinical workloads (87.3%), shift patterns of work (77.7%) and a lack of adequate trained staff members (77.3%) were the most commonly recognised barriers. Lack of availability of research staff to help with recruitment, particularly during busy times and out of hours (57%), was recognised as an important barrier, as these were noted to be times with excess patient flow but reduced staff, which resulted in missing out on eligible participants for recruitment.

An important regulatory hurdle identified by a high proportion of responders was Good Clinical Practice (GCP) training for clinical staff (69.6%). Arranging GCP training and encouraging clinical staff to attend was found to be very difficult. Rapid turnover of doctors and shift patterns of work made it difficult to have GCP-trained staff in every shift, again hindering recruitment of eligible patients.

The time and setting of consent seeking was identified as an important barrier (76%). There was a 20-minute window period for taking informed consent while the patient was receiving initial treatment in the emergency department or children’s assessment unit. Seeking consent from the parents of an ill child in an acute or emergency setting in 20 minutes was found to be very challenging by the clinical teams. Local clinical arrangements and choice of recruitment setting (52.1%) were highlighted as barriers; some sites recruiting from the paediatric wards reported that patients were not eligible for recruitment by the time they reached the ward and suggested recruiting patients in the accident and emergency department.

### Perceptions of PIs and research nurses with regard to facilitators and barriers to recruitment

The responses from the PIs and the research nurses were also analysed separately. The perceptions of PIs were different from those of research nurses and from overall responses in some respects. Motivation of the clinical team, their experience and training in providing information and seeking consent and communication between research team and parents were not recognised as facilitators by PIs. However, research experience of the clinical team was thought to boost recruitment.

The PIs did not see parents’ concerns about side effects of the drug or their anxiety related to their child taking experimental medicine as barriers, which may be explained by their experience and skills in communicating with parents. This group also did not find it difficult to approach patients for consent. Language and cultural barriers were not perceived to be important, and information provided to parents or families was not felt to be excessive or too complex. This group regarded consent seeking by senior doctors and nurses as a hindrance to recruitment.

The perceptions of research nurses were also different in some respects, compared to overall responses and PI responses. They recognised some additional factors as facilitators such as the presence of clinical equipoise, which was not identified in overall responses or by the PIs. This group perceived that recruitment was better if senior doctors and nurses sought consent. They also identified some additional barriers. The clinical team was thought to be lacking in research experience and motivation, which hindered recruitment. Research was not perceived to be important in routine clinical practice, and the local research culture was felt to be unhelpful. They identified additional practical constraints such as data collection and trial demands resulting from the study protocol being different from routine clinical practice. They felt that recruitment was hindered because MAGNETIC was a drug trial and parents were not familiar with the experimental medicine, resulting in a low consent rate.

There were also differences in opinion between PIs and research nurses in some of the domains as to whether they were facilitators or barriers. In 15 sites (55%) there was a difference of perception as to the impact of the experience of the research team on study success. Fifty-three percent of PIs perceived this as a facilitator, whereas 77% of the research nurses regarded this as a barrier. In 12 sites (46%) there was a difference in perception of the impact of senior clinicians and nurses seeking consent on ease of recruitment. Fifty-eight percent of PIs regarded this as a barrier, whereas 77% of the research nurses regarded this as a facilitator.

### Recruitment strategies or interventions that were applied at sites to improve recruitment

Free text responses were received from 108 participants on interventions or strategies that were applied to overcome the barriers to recruitment. The number and percentage of responders who identified each of the listed recruitment strategies are presented in Table 4. Having a designated research nurse was the most commonly reported intervention. Research nurses were found to be very helpful in all stages of recruitment, providing hands-on support to busy clinical teams, encouraging staff to participate and providing training. They were thought to be instrumental in bringing about a change in culture at sites and were described as ‘critical’, ‘essential to the success of the trial’ and ‘very effective’. One site attributed its success to appointment of a paediatric research nurse who was described to have ‘generated enthusiasm in the clinical team’ and ‘made protocol violations extremely unlikely through education and reminders’, leading to a ‘dramatic improvement in recruitment’.

**Table 4:** Recruitment strategies

Recruitment strategy	*n* (%)
Having a designated research nurse	27 (25)
Arranging GCP training and encouraging staff to attend	22 (20.4)
Trial-related teaching and training of staff	21 (19.4)
Trial publicity	11 (10.2)
Motivation and support provided by PIs and senior medical team	11 (10.2)
Regular communication and meetings with staff	9 (8.3)
Measures to improve availability of staff to recruit	5 (4.6)
Additional support during out of hours; extra staff, twilight nurses	5 (4.6)
Encouraging staff to be more involved in the trial	5 (4.6)
Repeated reminders to clinical staff	5 (4.6)
Clinical Trials Unit (CTU) support and involvement of trial coordinator	4 (3.7)
Incentives offered to staff	4 (3.7)
Nurse consent and prescribing	3 (2.7)
Simpler trial instructions and simpler paperwork	3 (2.7)
Weekly screening and ‘chasing’ medical staff for eligible patients missed	2 (1.9)

Arranging GCP training was the second most commonly reported strategy and was reported to be very effective. However, it was found to be challenging, ‘hard to maintain’ and difficult to train all doctors due to practical constraints such as heavy workloads, short-term sickness and a high rate of doctor turnover. Other interventions included teaching and training of staff and trial publicity measures, e.g. putting up posters and ‘aide memoires’ such as ‘Got a wheeze? Think MAGNETIC’. Motivation and support provided by senior medical staff, senior staff being available to help, regular reminders to staff, improved availability of doctors and nurses and additional support measures during out of hours, such as extra staff and twilight nurses and having adequate funding to support these measures, were all considered to be very helpful.

Recruitment and consent by senior and more experienced members of the team was felt to be very important, as ‘seeking consent in the acute setting where treatment needs to be initiated ASAP (as soon as possible)’ was thought to ‘put parents and clinical teams under pressure’. CTU support, having a dedicated trial manager and good relationships and effective communications between teams were found to be very effective. Other reported interventions included shorter and simpler trial instructions, simpler paperwork, reduced data collection and weekly screening and ‘chasing’ of medical staff for missing eligible patients.

## Discussion

The study findings endorse the various facilitators and barriers to recruitment to clinical trials that have been identified in the existing literature. Motivation of the clinical team, good communication skills, research experience of the PI and clinical team, good trial management, research nurse support, a positive research culture and effective communication between teams and with patients have been recognised as important factors that boost recruitment [4]. Time constraints of clinicians, heavy clinical workloads, shift patterns and training and staffing issues have been recognised as important hindrances to recruitment [8].

The clinical teams recognised parents’ apprehension about their child taking an experimental medicine and their concerns about the potential adverse effects as barriers, which have been previously described as important considerations for parents when deciding for their child to participate in clinical trials [9]. Paediatricians have been found to consider trial participation as an additional burden for parents. This is similar to the perception of clinical teams in our study who expressed discomfort in approaching patients for research [9], thereby reiterating the need for mentoring and providing training and support to clinical staff. An excess amount and complexity of trial information provided in the patient information leaflets has been previously criticised, similar to the findings in this survey.

This study highlights research nurse support and the presence of designated research teams, particularly out of hours, as very important. Another factor to note is GCP training, which has been described as a ‘massive hurdle’. Engaging doctors to undertake GCP training was found to be very difficult and described as ‘time consuming, boring and not a priority for busy clinicians’. The study also found some differences in the perceptions of PIs and research nurses with regard to certain facilitators and barriers to recruitment. This highlights the need to consider varying viewpoints within a research team so that appropriate measures can be taken to facilitate recruitment, and may be useful to bear in mind when planning future trials.

An important trial-specific barrier was seeking consent from the parents of an ill child in 20 minutes, which raises the issue of an option of deferred consent being available for paediatric trials in acute or emergency settings. The UK law incorporates a deferred consent process in emergency situations for minors [10] when treatment is required urgently, urgent action is required for the purposes of the trial, consent cannot be obtained prospectively and the procedure has been approved by the ethics committee. Deferred consent has been successfully used in a recent randomised controlled trial evaluating the role of impregnated central venous catheters (impregnated with antibiotics or with heparin) or a standard central venous catheter for prevention of blood stream infections in children (the CATCH trial) [11]. For children needing a central venous catheter placement as an emergency, a written parental consent was sought after randomisation to avoid delay in treatment. A postal survey [12] investigating parents’ views about deferred consent in a paediatric emergency setting showed that the majority of parents found it acceptable. However, the death of a child during a trial in which deferred consent has been used presents a complex situation. The authors highlight the need for further evidence to guide appropriate management in these cases.

This study highlights several important factors that affect recruitment to clinical trials. The strengths of our study include an electronic survey design, use of a comprehensive, evidence-based recruitment survey tool, a wide range of responders with different roles from sites with variable recruitment performance and a good representation of sites with high response rates from PIs and research nurses at each site. E-surveys are quicker, less expensive, can be sent to multiple responders at the same time, avoid interviewer bias and allow the responders to express their views freely and anonymously, even regarding sensitive issues [13].

Responses from a range of responders with different roles and sites with different recruitment performance increased the breadth of data gathered in terms of recruitment experience and perspectives, increasing the precision and generalisability of the results. A high response rate from the PIs and at least one research nurse from each site ensured equal representation of sites in overall responses, thereby ensuring generalisability and avoiding selection and non-respondent bias.

The study has some potential limitations. The survey questionnaire has the disadvantage of respondent non-response, misinterpretation of questions or selective responder bias. To overcome these limitations, the survey questionnaire was worded in a simple and clear language and piloted prior to use. The non-responders were sent frequent email reminders.

The overall response rate was low, though we obtained a good response rate from the PIs and research nurses. The denominator included all contacts whose email addresses were available from the delegation logs; thus, it is likely that not all contacts would have received the survey if their email addresses had changed and were different at the time the survey was conducted. There was a high likelihood of people changing jobs or rotating between different NHS trusts during the 2-year duration of the trial, particularly doctors in training, nursing staff and other junior doctors, resulting in a change in their email addresses and contact details. This was pre-empted during the planning stage of this study, and extra efforts were made to receive a response from the PI and at least one research nurse per site.

The results of the survey are based on subjective experiences of clinical staff responding to the survey questionnaire and descriptive analysis of responses. We tried to overcome this limitation by achieving a good representation of sites in overall responses and analysing the PI and at least one research nurse response at each site separately. This study presents the recruitment experience of the clinical teams recruiting to the trial. Understanding the perspective of other stakeholders such as parents, young people and families is also very important.

## Conclusions

This study explored the recruitment experience of various clinical teams recruiting to the MAGNETIC trial and identified important facilitators and barriers and information on strategies adopted by clinical teams to boost recruitment. The findings of the study can be generalised to other trials, particularly in the acute/emergency setting, as the study helped to identify both generic and trial-specific facilitators and barriers to recruitment and can be used to inform the design and conduct of future clinical trials.

The importance of having motivated and enthusiastic clinical teams, good communication skills and a positive research culture is emphasised. We recommend trialists to consider using study designs with simpler protocols that are comparable to routine clinical practice, with less restrictive inclusion criteria and less excessive data collection. Designated research nurses should be made available at sites to assist clinical staff with recruitment, particularly out of hours. This is particularly applicable to trials in the acute or emergency setting with heavy clinical workloads. The option of deferred consent should be considered for trials in the emergency setting. Clinical staff should be encouraged to undertake GCP training, and efforts should be made to motivate doctors and nursing staff to participate in research. Adequate training should be provided to doctors to enhance their confidence and skills in communicating with parents and families, seeking informed consent and allaying their concerns about the trial or experimental medicine. Patient information leaflets should be kept short, and information should be provided in a simple and clear manner. Trial publicity measures such as posters and banners should be put up to maintain awareness of trials among staff and patients. Central trial management support should be provided, and efforts should be made to ensure effective communication between clinical teams at various sites.

## Appendix

Table 5 shows data on overall responses and responses from PIs and research nurses on facilitators and barriers to recruitment.

**Table 5:** Presentation of the recruitment survey questionnaire used to collect data on facilitators and barriers to recruitment to the MAGNETIC trial

Factor	Facilitator, *n* (%)			Not applicable, *n* (%)			Barrier, *n* (%)		
	O	PI	RN	O	PI	RN	O	PI	RN
Funding	55 (33.0)	14 (43.8)	13 (43.3)	88 (52.7)	13 (40.6)	9 (30.0)	24 (14.4)	5 (15.6)	8 (26.7)
Trial design	71 (42.5)	12 (37.5)	13 (43.3)	33 (19.8)	5 (15.6)	5 (16.7)	63 (37.8)	15 (46.9)	12 (40.0)
Patient inclusion criteria	96 (57.5)	20 (64.5)	20 (66.7)	25 (15.0)	4 (12.9)	3 (10.0)	46 (27.6)	7 (22.6)	7 (23.3)
MAGNETIC being a drug trial	50 (30.0)	10 (31.3)	6 (20.7)	49 (29.3)	17 (53.1)	5 (17.2)	68 (40.7)	5 (15.6)	18 (62.1)
Study protocol compared to clinical practice	61 (36.6)	13 (40.6)	12 (40.0)	28 (16.8)	9 (28.1)	1 (3.3)	78 (46.7)	10 (31.3)	17 (56.7)
Clinical equipoise	69 (42.8)	13 (41.9)	16 (53.3)	72 (44.7)	16 (51.6)	10 (33.3)	20 (12.4)	2 (6.5)	4 (13.3)
Previous feasibility assessment	51 (31.3)	9 (28.1)	11 (39.3)	99 (60.7)	20 (62.5)	15 (53.6)	13 (8.0)	3 (9.4)	2 (7.1)
Previous pilot trial	52 (32.1)	10 (32.3)	11 (39.3)	104 (64.2)	19 (61.3)	16 (57.1)	6 (3.7)	2 (6.5)	1 (3.6)
Publicity by the trial team	103 (62.9)	19 (59.4)	22 (75.9)	43 (26.2)	12 (37.5)	1 (3.4)	18 (10.9)	1 (3.1)	6 (20.7)
External publicity	36 (21.8)	6 (18.8)	6 (20.7)	110 (66.7)	24 (75.0)	17 (58.6)	19 (11.6)	2 (6.3)	6 (20.7)
Trial management	101 (62.0)	19 (59.4)	24 (82.8)	42 (25.8)	9 (28.1)	2 (6.9)	20 (12.3)	4 (12.5)	3 (10.3)
Protocol amendments	49 (29.5)	5 (15.6)	12 (40.0)	90 (54.2)	20 (62.5)	14 (46.7)	27 (16.3)	7 (21.9)	4 (13.3)
Seasonal variation	40 (24.0)	4 (12.5)	10 (33.3)	40 (24.1)	8 (25.0)	2 (6.7)	86 (51.8)	20 (62.5)	18 (60.0)
Site-level factors									
Time to open up site	33 (20.0)	5 (15.6)	6 (20.0)	75 (45.5)	9 (28.1)	11 (36.7)	57 (34.5)	18 (56.3)	13 (43.3)
Recruitment target	62 (37.8)	11 (34.4)	13 (46.4)	59 (36.0)	12 (37.5)	9 (32.1)	43 (26.2)	9 (28.1)	6 (21.4)
Time to complete administrative work related to the trial	24 (14.3)	3 (9.4)	7 (23.3)	32 (19.0)	9 (28.1)	5 (16.7)	112 (66.6)	20 (62.5)	18 (60.0)
Number of trained staff	26 (15.5)	4 (12.5)	3 (10.0)	12 (7.1)	2 (6.3)	2 (6.7)	130 (77.3)	26 (81.3)	25 (83.3)
Local clinical arrangements	43 (26.1)	7 (22.6)	6 (20.0)	36 (21.8)	7 (22.6)	3 (10.0)	86 (52.1)	17 (54.8)	21 (70.0)
Choice of recruitment setting	72 (43.9)	12 (37.5)	13 (43.3)	39 (23.8)	9 (28.1)	2 (6.7)	53 (32.3)	11 (34.4)	15 (50.0)
Good Clinical Practice (GCP) training	33 (19.8)	2 (6.3)	4 (13.3)	18 (10.8)	3 (9.4)	0 (0)	116 (69.6)	27 (84.4)	26 (86.7)
Data collection process	44 (26.5)	10 (31.3)	6 (20.0)	44 (26.5)	14 (43.8)	4 (13.3)	78 (47.0)	8 (25.0)	20 (66.7)
Competing local research projects	23 (13.8)	2 (6.3)	3 (10.0)	116 (69.5)	25 (78.1)	19 (63.3)	28 (16.8)	5 (15.6)	8 (26.7)
Local research culture	65 (39.2)	13 (40.6)	7 (23.2)	32 (19.3)	7 (21.9)	1 (3.3)	69 (41.6)	12 (37.5)	22 (73.3)
Patient factors									
Consent rate	48 (29.1)	6 (18.8)	8 (26.7)	39 (23.6)	11 (34.4)	5 (16.7)	78 (47.2)	15 (46.9)	17 (56.7)
Familiarity with experimental treatment	52 (31.3)	7 (21.9)	7 (23.3)	44 (26.5)	13 (40.6)	5 (16.7)	70 (42.1)	12 (37.5)	18 (60.0)
Parent's attitude towards their child taking experimental medicine or placebo	26 (15.6)	3 (9.4)	5 (17.2)	45 (27.1)	15 (46.9)	6 (20.7)	95 (57.2)	14 (43.8)	18 (62.1)
Parent's preference for a particular treatment	16 (9.6)	0 (0.0)	3 (10.0)	92 (55.4)	26 (81.3)	14 (46.7)	58 (34.9)	6 (18.8)	13 (43.3)
Parent's concerns about side effects of new drug	12 (7.2)	1 (3.1)	1 (3.3)	46 (27.5)	16 (50.0)	6 (20.0)	109 (65.3)	15 (46.9)	23 (76.7)
Duration of trial and follow-up	59 (35.5)	11 (34.4)	12 (41.4)	62 (37.3)	17 (53.1)	7 (24.1)	45 (27.1)	4 (12.5)	10 (34.5)
Treatment choice by random allocation	42 (25.2)	7 (21.9)	7 (23.3)	80 (47.9)	21 (65.6)	13 (43.3)	45 (27.1)	4 (12.5)	10 (33.3)
Additional trial investigations	15 (9.1)	3 (9.4)	2 (6.7)	111 (67.3)	26 (81.3)	17 (56.7)	39 (23.6)	3 (9.4)	11 (36.7)
Additional travel and extra costs	9 (5.4)	3 (9.4)	2 (6.7)	146 (88)	27 (84.4)	27 (90.0)	11 (6.6)	2 (6.3)	1 (3.3)
Intervention available only in the trial	34 (20.5)	5 (15.6)	10 (33.3)	108 (65.1)	25 (78.1)	16 (53.3)	24 (14.4)	2(6.3)	4 (13.3)
Communication between research team and parents	83 (50.1)	11 (34.4)	21 (70.0)	55 (33.1)	19 (59.4)	3 (10.0)	28 (16.8)	2(6.3)	6 (20.0)
Clinician influence	73 (44.0)	9 (28.1)	14 (46.7)	67 (40.4)	19 (59.4)	8 (26.7)	26 (15.6)	4 (12.5)	8 (26.7)
Language or cultural barriers	11 (6.1)	1 (3.1)	1 (3.3)	72 (43.1)	20 (62.5)	13 (43.3)	84 (50.3)	11 (34.4)	16 (53.3)
Clinical team factors									
Research experience of clinical team	71 (42.6)	17 (53.1)	6 (20.0)	16 (9.6)	3 (9.4)	1 (3.3)	80 (47.9)	12 (37.5)	23 (76.7)
Presence of designated research nurse/practitioner	113 (68.1)	22 (68.8)	19 (65.5)	17 (10.2)	2 (6.3)	2 (6.9)	36 (21.6)	8 (25.0)	8 (27.6)
Availability of designated research team	97 (58.5)	21 (65.6)	18 (60.0)	24 (14.5)	5 (15.6)	0 (0)	45 (27.0)	6 (18.8)	12 (40.0)
Availability of research staff out of hours	30 (18)	5 (15.6)	6 (20.0)	42 (25.1)	8 (25.0)	5 (16.7)	95 (57.0)	19 (59.4)	19 (63.3)
Shift patterns of work	15 (9)	3 (9.4)	1 (3.3)	22 (13.3)	2 (6.3)	4 (13.3)	129 (77.7)	27 (84.4)	25 (83.3)
Motivation of clinical team	89 (53.6)	16 (50.0)	12 (40.0)	14 (8.4)	4 (12.5)	1 (3.3)	63 (37.9)	12 (37.5)	17 (56.7)
Clinical workload	11 (6.6)	2 (6.3)	1 (3.3)	10 (6.0)	2 (6.3)	1 (3.3)	146 (87.3)	28 (87.5)	28 (93.3)
Perceived importance of research generally in clinical practice	76 (45.8)	14 (43.8)	8 (26.7)	24 (14.5)	3 (9.4)	3 (10.0)	66 (39.8)	15 (46.9)	19 (63.3)
Perceived importance of the particular research question	99 (60.1)	20 (62.5)	16 (53.3)	34 (20.6)	6 (18.8)	6 (20.0)	32 (19.4)	6 (18.8)	8 (26.7)
Communication skills of clinical team	116 (70.3)	24 (75)	19 (63.3)	26 (15.8)	7 (21.9)	2 (6.7)	23 (13.9)	1 (3.1)	9 (30.0)
Clinician preference for particular treatment	36 (22.0)	5 (16.1)	7 (24.1)	110 (67.5)	23 (74.2)	19 (65.5)	17 (10.4)	3 (9.7)	3 10.3)
Clinician attitude to involving patients in research	101 (60.9)	18 (56.3)	18 (60.0)	35 (21.1)	9 (28.1)	5 (16.7)	30 (18.0)	5 (15.6)	7 (23.3)
Difficulty in approaching patients for consent	16 (9.6)	3 (9.4)	4 (13.3)	61 (36.5)	16 (50.0)	11 (36.7)	90 (53.9)	13 (40.6)	15 (50.0)
Information and consent-related factors									
Amount and complexity of trial information provided	53 (31.9)	10 (31.3)	11 (36.7)	30 (18.1)	11 (34.4)	2 (6.7)	83 (50.0)	11 (34.4)	17 (56.7)
Clarity in presentation of trial information	97 (58.4)	19 (59.4)	20 (66.7)	25 (15.1)	8 (25.0)	2 (6.7)	44 (26.5)	5 (15.6)	8 (26.7)
Social and emotional dynamics of trial discussion	35 (21.0)	7 (21.9)	10 (33.3)	78 (47.0)	20 (62.5)	8 (26.7)	53 (31.9)	5 (15.6)	12 (40.0)
Time and setting of consent seeking	18 (10.8)	2 (6.3)	4 (13.3)	22 (13.2)	4 (12.5)	3 (10.0)	127 (76.0)	26 (81.3)	23 (76.7)
Senior doctors and nurses seeking consent	75 (45.3)	8 (25.8)	16 (53.3)	25 (15.1)	5 (16.1)	2 (6.7)	66 (39.8)	18 (58.1)	12 (40.0)
Experience and training of clinical team seeking consent	84 (50.4)	12 (37.5)	16 (53.3)	27 (16.2)	8 (25.0)	0 (0)	56 (33.6)	12 (37.5)	14 (46.7)
Study team factors									
Motivation of MAGNETIC study team at site	131 (78.9)	25 (80.6)	22 (73.3)	14 (8.4)	3 (9.7)	1 (3.3)	21 (12.6)	3 (9.7)	7 (23.3)
Communication and coordination between study team members at site	123 (74.5)	24 (77.4)	22 (73.3)	25 (15.2)	6 (19.4)	2 (6.7)	17 (10.2)	1 (3.2)	6 (20.0)
Communication and coordination between study team at site and CTU	103 (62.1)	19 (61.3)	27 (90.0)	49 (29.5)	9 (29)	3 (10.0)	14 (8.4)	3 (9.7)	0 (0)
Research experience of PI and study team members at site	105 (63.3)	19 (61.3)	21 (70.0)	38 (22.9)	7 (22.6)	3 (10.0)	23 (13.8)	5 (16.1)	6 (20.0)

## Additional file

Additional file 1: Presents the recruitment survey questionnaire that was used to gather information on facilitators and barriers and strategies applied to boost recruitment. (DOCX 55 kb)

## Abbreviations

ASAP: As soon as possible
CTU: Clinical Trials Unit
GCP: Good Clinical Practice
MCRN: Medicines for Children Research Network
NIHR: National Institute of Health Research
PI: Principal Investigator
UK: United Kingdom
