# Supplementary material for: A survey of facilitators and barriers to recruitment to the MAGNETIC trial
Source: Trials. 2016 Dec 23;17:607. doi: 10.1186/s13063-016-1724-3 (PMC5180395; doi:10.1186/s13063-016-1724-3)
Supplement: Additional file 1: — Presents the recruitment survey questionnaire that was used to gather information on facilitators and barriers and strategies applied to boost recruitment. (DOCX 55 kb) [file 13063_2016_1724_MOESM1_ESM.docx]

## Additional file 1: MAGNETIC recruitment survey

**1) Please enter your ID number**

**2) Please indicate your role with regards to recruitment to MAGNETIC**

Site lead/PI

Medical practitioner

Research nurse

Other

**Please describe your role in relation to recruitment to MAGNETIC**

**3) Which site/hospital were you recruiting from?**

**4) Have you been involved with MAGNETIC for the whole trial period?**

Yes

No

**Was this during**

setup/early recruitment period

once trial established at site

**How long were you involved in recruiting for MAGNETIC? (approximately, in months)**

**5) Listed below are trial specific factors that commonly affect recruitment. Please indicate whether a listed factor was a facilitator or barrier to recruitment to MAGNETIC and rate them from -3 to +3 as below:
-3 strong barrier
-2 intermediate barrier
-1 weak barrier
0 not applicable
+1 weak facilitator
+2 intermediate facilitator
+3 strong facilitator**

|  | **-3** | **-2** | **-1** | **0** | **+1** | **+2** | **+3** |
| --- | --- | --- | --- | --- | --- | --- | --- |
| Funding |  |  |  |  |  |  |  |
| Trial design |  |  |  |  |  |  |  |
| Patient inclusion criteria |  |  |  |  |  |  |  |
| MAGNETIC being a drug trial |  |  |  |  |  |  |  |
| Study protocol compared to clinical practice |  |  |  |  |  |  |  |
| Clinical equipoise |  |  |  |  |  |  |  |
| Previous feasibility assessment |  |  |  |  |  |  |  |
| Previous pilot trial |  |  |  |  |  |  |  |
| Publicity by the trial team |  |  |  |  |  |  |  |
| External publicity |  |  |  |  |  |  |  |
| Trial management |  |  |  |  |  |  |  |
| Protocol amendments |  |  |  |  |  |  |  |
| Seasonal variation |  |  |  |  |  |  |  |

**6) Listed below are site specific factors that commonly affect recruitment. Please indicate whether a listed factor was a facilitator or barrier to recruitment to MAGNETIC and rate them from -3 to +3 as below:
-3 strong barrier
-2 intermediate barrier
-1 weak barrier
0 not applicable
+1 weak facilitator
+2 intermediate facilitator
+3 strong facilitator**

|  | **-3** | **-2** | **-1** | **0** | **+1** | **+2** | **+3** |
| --- | --- | --- | --- | --- | --- | --- | --- |
| Time to open up site |  |  |  |  |  |  |  |
| Recruitment target |  |  |  |  |  |  |  |
| Time to complete administrative work related to the trial |  |  |  |  |  |  |  |
| Number of trained staff |  |  |  |  |  |  |  |
| Local clinical arrangements |  |  |  |  |  |  |  |
| Choice of recruitment setting |  |  |  |  |  |  |  |
| GCP training |  |  |  |  |  |  |  |
| Data collection process |  |  |  |  |  |  |  |
| Competing local research projects |  |  |  |  |  |  |  |
| Local research culture |  |  |  |  |  |  |  |

**7) Listed below are patient specific factors that commonly affect recruitment. Please indicate whether a listed factor was a facilitator or barrier to recruitment to MAGNETIC and rate them from -3 to +3 as below:
-3 strong barrier
-2 intermediate barrier
-1 weak barrier
0 not applicable
+1 weak facilitator
+2 intermediate facilitator
+3 strong facilitator**

|  | **-3** | **-2** | **-1** | **0** | **+1** | **+2** | **+3** |
| --- | --- | --- | --- | --- | --- | --- | --- |
| Consent rate |  |  |  |  |  |  |  |
| Familiarity with experimental treatment |  |  |  |  |  |  |  |
| Parent's attitude towards their taking experimental medicine or placebo |  |  |  |  |  |  |  |
| Parent's preference for a particular treatment |  |  |  |  |  |  |  |
| Parent's concerns about side effects of new drug |  |  |  |  |  |  |  |
| Duration of trial and follow up |  |  |  |  |  |  |  |
| Treatment choice by random allocation |  |  |  |  |  |  |  |
| Additional trial investigations |  |  |  |  |  |  |  |
| Additional travel and extra costs |  |  |  |  |  |  |  |
| Intervention available only in the trial |  |  |  |  |  |  |  |
| Communication between research team and parents |  |  |  |  |  |  |  |
| Clinician influence |  |  |  |  |  |  |  |
| Language or cultural barriers |  |  |  |  |  |  |  |

**8) Listed below are clinical team factors that commonly affect recruitment. Please indicate whether a listed factor was a facilitator or barrier to recruitment to MAGNETIC and rate them from -3 to +3 as below:
-3 strong barrier
-2 intermediate barrier
-1 weak barrier
0 not applicable
+1 weak facilitator
+2 intermediate facilitator
+3 strong facilitator**

|  | **-3** | **-2** | **-1** | **0** | **+1** | **+2** | **+3** |
| --- | --- | --- | --- | --- | --- | --- | --- |
| Research experience of clinical team |  |  |  |  |  |  |  |
| Presence of designated research nurse/practitioner |  |  |  |  |  |  |  |
| Availability of designated research team |  |  |  |  |  |  |  |
| Availability of research staff out of hours |  |  |  |  |  |  |  |
| Shift patterns of work |  |  |  |  |  |  |  |
| Motivation of clinical team |  |  |  |  |  |  |  |
| Clinical workload |  |  |  |  |  |  |  |
| Perceived importance of research generally in clinical practice |  |  |  |  |  |  |  |
| Perceived importance of the particular research question |  |  |  |  |  |  |  |
| Communication skills of clinical team |  |  |  |  |  |  |  |
| Clinician preference for particular treatment |  |  |  |  |  |  |  |
| Clinician attitude to involving patients in research |  |  |  |  |  |  |  |
| Difficulty in approaching patients for consent |  |  |  |  |  |  |  |

**9) Listed below are Information and consent related factors that commonly affect recruitment. Please indicate whether a listed factor was a facilitator or barrier to recruitment to MAGNETIC and rate them from -3 to +3 as below:
-3 strong barrier
-2 intermediate barrier
-1 weak barrier
0 not applicable
+1 weak facilitator
+2 intermediate facilitator
+3 strong facilitator**

|  | **-3** | **-2** | **-1** | **0** | **+1** | **+2** | **+3** | |
| --- | --- | --- | --- | --- | --- | --- | --- | --- |
| Amount and complexity of trial information provided |  |  |  |  |  |  |  |  |
| Clarity in presentation of trial information |  |  |  |  |  |  |  |  |
| Social and emotional dynamics of trial discussion |  |  |  |  |  |  |  |  |
| Time and setting of consent seeking |  |  |  |  |  |  |  |  |
| Senior doctors and nurses seeking consent |  |  |  |  |  |  |  |  |
| Experience and training of clinical team seeking consent |  |  |  |  |  |  |  |  |

**10) Listed below are study team factors that commonly affect recruitment. Please indicate whether a listed factor was a facilitator or barrier to recruitment to MAGNETIC and rate them from -3 to +3 as below:
-3 strong barrier
-2 intermediate barrier
-1 weak barrier
0 not applicable
+1 weak facilitator
+2 intermediate facilitator
+3 strong facilitator**

|  | **-3** | **-2** | **-1** | **0** | **+1** | **+2** | **+3** |
| --- | --- | --- | --- | --- | --- | --- | --- |
| Motivation of MAGNETIC study team at site |  |  |  |  |  |  |  |
| Communication and coordination between study team members at site |  |  |  |  |  |  |  |
| Communication and coordination between study team at site and CTU |  |  |  |  |  |  |  |
| Research experience of PI and study team members at site |  |  |  |  |  |  |  |

**11) What interventions or strategies were applied to overcome any hurdles identified in previous questions and how effective were these?**

**12) How would you organise MAGNETIC differently to improve recruitment? Please include additional comments, if any**
